# Local adaptation and validation of a transdiagnostic risk calculator for first episode psychosis using mental health patient records

**DOI:** 10.3389/fpsyt.2025.1584719

**Published:** 2025-07-22

**Authors:** Elizabeth Ford, James Stone, Dominic Oliver, Benjamin Fell, Gloria Roque, Sam Robertson, Paolo Fusar-Poli, Kathryn Greenwood

**Affiliations:** 1 Department of Primary Care and Public Health, Brighton and Sussex Medical School, University of Brighton and University of Sussex, Brighton, United Kingdom; 2 Department of Clinical Neuroscience, Brighton and Sussex Medical School, University of Brighton and University of Sussex, Brighton, United Kingdom; 3 Sussex Partnership National Health Service (NHS) Foundation Trust, Worthing, United Kingdom; 4 Department of Psychiatry, University of Oxford, Oxford, United Kingdom; 5 National Institute of Health Research (NIHR) Oxford Health Biomedical Research Centre, Oxford, United Kingdom; 6 OPEN Early Detection Service, Oxford Health NHS Foundation Trust, Oxford, United Kingdom; 7 Akrivia Health, Oxford, United Kingdom; 8 Department of Brain and Behavioral Sciences, University of Pavia, Pavia, Italy; 9 Early Psychosis: Interventions and Clinical-Detection (EPIC) Lab, Department of Psychosis Studies, Institute of Psychiatry, Psychology and Neuroscience, King’s College London, London, United Kingdom; 10 OASIS Service, South London and Maudsley NHS Foundation Trust, London, United Kingdom; 11 Department of Psychiatry and Psychotherapy, Ludwig-Maximilian-University, Munich, Germany; 12 School of Psychology, University of Sussex, Brighton, United Kingdom

**Keywords:** psychosis, risk assessment, natural language processing, electronic health records, mental health care, at risk mental state

## Abstract

**Background:**

Few at-risk adults are identified by specialized services prior to the development of a first episode of psychosis. A transdiagnostic risk calculator, predicting psychosis using electronic health record (EHR) data, was developed in London, UK to identify patients at risk, using structured data and 14 natural language processing (NLP)-derived symptom and substance use concepts. We report the adaptation and internal validation of this risk calculator in a Southeast England region.

**Methods:**

In a retrospective cohort study using EHR patient notes we identified individuals accessing mental healthcare in Southeast England (Nov-1992 to Jan-2023) who received a primary diagnosis of a non-psychotic or non-organic mental disorder. We developed new machine-learning NLP algorithms for diagnosis, symptom and substance use concepts by fine-tuning existing open-source transformer models. Baseline and outcome coded diagnoses were supplemented with NLP-derived diagnosis data. Cox regression was used to predict psychosis and prior weights were applied; discrimination (Harrell’s C) was assessed.

**Results:**

Nearly all NLP concepts achieved an F1-measure of accuracy above 0.8 following development. In an analysis sample of 63,922 patients with complete data, the risk calculator had acceptable but lower accuracy in Southeast England (Harrell’s C 0.71) compared to the London benchmark (Harrell’s C 0.85).

**Conclusions:**

The risk calculator performed similarly in Southeast England to other external validation studies, discriminating acceptably, suggesting that this calculator may be adapted successfully for new patient populations, services and geographic areas. Differences in accuracy may be due to different cultures of data capture, different NLP approaches, or differences in the patient cohort.

## 1 Introduction

The coastal Southeast region of England has high proportions of young adults with emotional and substance use problems, which are risk factors for the onset of psychosis (1). However, 95% of at risk young people referred to secondary mental health services are not recognized as ‘at risk’ and potentially fall through gaps in services (2, 3). Our own work in the EYE-2 study has shown that 40% of patients on average (and up to 90% in some areas) referred to Early Intervention in Psychosis (EIP) services nationally are not accepted, due in part to stringent application of criteria (4). Prevention, early intervention and use of data to reduce inequalities and improve services are key priorities for the new National Health Service integrated care systems in coastal Southeast England (5).

There are many opportunities to use psychiatric and other electronic health records (EHRs) to develop effective clinical prediction models for predicting mental health outcomes and identifying at-risk groups across the age spectrum (6). Research has shown that certain mood, neurotic and physical symptoms (e.g. sleep disturbance, poor appetite), as well as substance use, are common in EHRs before a first episode of psychosis (7). However, the quality of data is variable and extracting information in an accurate, scalable way, presents a number of challenges, due to the complex infrastructures for storing and coding of data in mental health EHRs, and the volume of unstructured, missing and duplicated data (8). Mental health trust EHRs hold 70% of actionable health data as unstructured case-note text (9), meaning that innovative analytic methods are needed to derive patient information in an accurate, structured, analyzable way which can then support targeted interventions.

The application of Natural Language Processing (NLP) and machine learning tools have provided the opportunity to predict more accurately those at greatest risk of developing mental disorders, such as psychosis, in other United Kingdom settings. A transdiagnostic risk calculator for psychosis was developed in a London dataset in South London and Maudsley NHS Foundation Trust (SLaM) and has now been tested in multiple settings within and outside the UK, using largely structured data (index diagnosis, age, gender and ethnicity) with performance indicated by Harrell’s Concordance (C) index ranging 0.73-0.79 (3, 10, 11) (see methods for explanation of Harrell’s C). A recent refinement, adding a further 14 emotional, symptom and substance use factors extracted from case notes using NLP increased the precision of the calculator, and discrimination performance was good in external validation (Harrell’s C 0.85) (12). Recent studies have begun to replicate these risk calculators in other healthcare systems (10, 11, 13), to explore clinicians’ use and responses to these predictions (14, 15), and to implement them in clinical practice (16), including a feasibility study in our own region.

The current study aimed to adapt and internally validate the SLaM transdiagnostic risk calculator for psychosis within data from a mental health service in Southeast England, reproducing the model in data curated via the Akrivia Clinical Record Interactive Search (CRIS) system (17), and evaluating the model’s performance to identify those patients who are at significant risk of developing psychosis in the region. We evaluated both the original structured data calculator and the extended calculator with 14 additional signs and symptoms taken from clinic notes.

This paper reports specifically on the following objectives:

1. Develop NLP machine learning algorithms to extract diagnosis, symptom and substance use data from case notes in local EHRs to ensure predictor availability for the transdiagnostic risk calculator.
2. Internally validate performance of the original and NLP-symptom enriched transdiagnostic risk calculator in the Southeast England dataset.

This work forms part of a wider project which aims to examine the feasibility of using the calculator in live prospective patient data to identify current patients who are at risk of developing psychosis in Southeast England, and provide a targeted, coproduced intervention, refined from the EYE-2 project (18, 19), to improve offers and uptake of support and mental health outcomes.

## 2 Methods

### 2.1 Design

We conducted a retrospective cohort study using routinely collected data from EHRs, structuring free text clinical data using natural language processing. This study is reported in accordance with the REporting of studies Conducted using Observational Routinely-collected health Data (RECORD) statement (20) (**Supplementary File 1**). No protocol was published or registered.

### 2.2 Data source

Data were drawn from an NHS mental healthcare service (“trust”) which is the primary healthcare provider of both inpatient and outpatient mental health care in two of the counties on the southeast coast of England. This area has a GP-registered population of 1.8 million. The counties have rural, urban and coastal areas with mostly lower deprivation than the national average, although some towns have pockets of very high deprivation (amongst the 20% most deprived in England), particularly in coastal towns. There is lower incidence of psychosis in this region in comparison to London (estimated with Psymaptic [http://www.psymaptic.org/] (Kirkbride et al., 2013)). The trust offers mental health, learning disability, neurodevelopmental, and dementia services. Of note, for the comparison with SLaM services, this region does not offer substance use services, which are found in SLaM. No data linkage was performed.

The trust has a contract with Akrivia Health Ltd to provide data curation services. The Akrivia CRIS system is a system for processing English electronic clinical notes, de-identifying them, and extracting and structuring relevant data for clinical, audit and research purposes from clinical text using NLP. It is being implemented in 20 mental health trusts across England. Extracted data related to patients who presented to or were currently being seen in mental health services from 5^th^ March 2008 when the electronic record system was adopted in the trust. Importing of patients’ historical structured diagnosis data means that some patients’ data goes back as far as 1992. A patient opt-out mechanism was in place.

### 2.3 Data governance and ethics approvals

Data held in the Akrivia system is protected by strict information security and by law. The database is a REC approved Research Database including an integrated governance model (IRAS ID 344145). Projects are reviewed and approved by a local oversight committee at each participating healthcare organization, comprised of clinicians, patients, and governance specialists.

Data about the study population is only available to researchers who are working with the mental health trust on approved projects. This project was approved by the Local Trust CRIS Oversight Committee and the London-Dulwich Research Ethics Committee (Ref: 21/LO/0872), and a DCB160 clinical safety review and DPIA were completed as part of the overarching project.

### 2.4 NLP development

The NLP solution used for this project was based on Google’s Bidirectional Encoder Representations from Transformers (BERT) model, a bidirectional Transformer pretrained on a vast text dataset with the key innovation of using self-attention [for more information on BERT’s original development see: 14]. NLP development occurred in late 2021.

Our implementation of BERT followed a three-step process:

1. Named Entity Recognition (NER): Identification and extraction of specific words or phrases embedded in a sentence and identification of it as one of the predefined entities.
2. Post-processing including ontology mapping: A mapping framework to clean and link extracted entities to standardized labels.
3. Contextual Classification: Categorization of extracted entities to specific classes based on its surrounding context and meaning.

For NER tasks, the pretrained BERT model was fine-tuned with an extensive training dataset of human-annotated clinical notes, which covered 5 concepts: medication, dosage, diagnosis, signs and symptoms (also called “patient experience”), and substance use. The annotation schema used to mark up clinical data with which to fine-tune the BERT models was overseen by a panel of clinical advisors and defined in collaboration with other HCO data partners to ensure clinical validity and research value. A team of 6 clinically-trained annotators marked up 13960 annotations for training the BERT model, across the 5 concepts, ranging from 936 for substance use to 7349 for signs and symptoms. Each annotation was marked by a single annotator, no inter-annotator agreement was calculated. To assess performance of the NER models, model output was compared to test sets annotated by the same team. For each concept the test set comprised of between 182 (substance use) and 1525 (signs and symptoms) annotations. Precision, recall and F1 measure were calculated for each concept.

Once entities were extracted, post-processing was carried out to prepare the data. This stage ensured that extractions were accurate by removing easily identified junk outputs, transforming data into categorical/numeric format, and mapping NLP outputs to additional data sources e.g., drug ontologies or Observational Medical Outcomes Partnership (OMOP; which includes ICD-10 code) vocabularies, by which they can be categorized or pseudo-coded. Only extractions that exactly matched terms within these ontologies were retained, ensuring consistency and reducing potential errors in classification. Furthermore, this ontology-based mapping eliminated the need to retain identifiable free-text extractions, as only structured, predefined terms were used.

The final step was running these post processed extractions through a contextual classification model. On this stage, extractions were categorized based on classes that have been pre-defined using an annotation schema: a structured framework of rules designed to capture the semantic meaning of clinical entities which is the basis of how the classifier model was trained. To build a robust schema, identification of key contextual features and their presence was analyzed for each clinical entity. The schema design varied depending on the clinical entity but primarily focused on extracting three key semantic features: negation (whether something occurred), temporality (when something occurred), and experiencer (who is experiencing it). In clinical text, these contextual features were often found to be interdependent and subject to interpretations. To address this complexity, we developed a single feature, ‘status’ which simultaneously classified negation, temporality, and experiencer. The status feature classified diagnoses into four distinct classes:

1. “Has” (Present Diagnosis): If sentence indicates that experiencer has the current diagnosis, it is classified under “has”.
o Example*”The patient has ongoing diabetes.”*
2. “Had” (Past Diagnosis): If an experiencer’s condition is described as in the past, the classifier assigns it to “had.”
o Example: *“Past history of schizophrenia.”*
3. “Does not have” (No Diagnosis): If the sentence explicitly states that the experiencer does not have the condition, the classifier assigns it to “does not have.”
o Example: *“There is no evidence of bipolar disorder.”*
o Example: *“The patient denies having depression.”*
4. “Could have” (Uncertain/Possible Diagnosis): If the diagnosis is uncertain, probable, or under discussion, the classifier categorizes it as “could have.”
o Example: *“It is unclear if the patient has COVID-19.”*

While the status attribute effectively classified most clinical entities by itself, additional linguistic complexities arise in diagnosis on clinical notes, particularly regarding family history mentions. Since many clinical notes contain references to diagnoses in family members, the status attribute alone could not always determine whether a diagnosis applies to the patient or someone else. An additional experiencer attribute was introduced to address this which classified diagnoses into two distinct classes:

1. “Patient”: The diagnosis applies to the patient.
o Example*”The patient has ongoing diabetes.”*
2. “Other”: The diagnosis refers to someone other than the patient, such as a family member.
o Example*”Family history: Father has schizophrenia.”*

The rules within the schema relied on linguistic cues to distinguish between conditions that are currently active, resolved, negated, or uncertain. These complex annotation rules were incorporated in the training and validation dataset to fine-tune the model. A semi-automated, human-in-the-loop annotation process was employed, integrating expert review through a group consensus of three team members facilitating the refinement of annotation rules and classification labels. An independent, unseen test set was curated to evaluate model performance. The test set was constructed by a consensus of three different team members who employed a pseudo-random selection process to categorize sentences. This was to ensure comprehensive representation of different sentence structures where common sentence types were over-represented in the test set compared to uncommon sentence types. Context classification was applied to NLP-derived diagnosis extractions, but only mention-level data on signs and symptoms was included.

### 2.5 Concept specification

To generate the structured dataset for analysis in this project, we specified a set of target concepts from both coded data and outputs produced by the Named Entity Recognition (NER) model (see **Supplementary Table 1** for list of concepts). These included the diagnosis concepts which formed both the baseline and outcome variables in the model, and the 14 signs, symptoms and substance use concepts which were used in the extended NLP model in SLaM (12).

### 2.6 Onboarding and exploring Southeast England data

Post-processed outputs from the NER model deployment were then uploaded to the project workspace for linkage with the core platform outputs by the project data science team.

### 2.7 Study sample

Previous work in SLaM data explored accuracy of an “original model” and a “refined model using NLP predictors” (13). The original model used three categorical baseline predictor variables (2), these were: ICD-10 cluster diagnosis (acute and transient psychotic disorders, substance use disorders, bipolar mood disorders, non-bipolar mood disorders, anxiety disorders, personality disorders, developmental disorders, childhood/adolescence onset disorders, physiological syndromes or intellectual disabilities; see **Supplementary Table 2** for a full list of ICD codes), gender (either male or female), and ethnicity (as Black, White, Asian, Mixed or Other) as well as age as a continuous variable.

Inclusion criteria in our study were matched to this; patients of any age needed a baseline diagnosis of a non-organic, non-psychotic disorder or an “acute and transient” psychotic disorder, and their record needed to include age, self-reported gender (in binary form) and ethnicity. See **Supplementary Table 2** for a full list of ICD codes used. Baseline diagnoses occurred between 1st November 1992 and 2^nd^ January 2023. Patients needed to have at least 3 months (91 days) of follow up to be included. They were excluded if they had a psychosis diagnosis recorded within 3 months of baseline diagnosis, as this may have reflected initial misdiagnosis as opposed to transition.

The outcome variable was any psychosis diagnosis made >3 months after the initial baseline diagnosis. This was to exclude acute and transient psychotic diagnoses (which were predictors), and to ensure that the psychosis diagnosis was a new diagnosis, not something that was co-occurring at baseline rather than a new onset disorder. These included schizophrenia spectrum psychoses (schizophrenia [ICD code: F20.x], schizoaffective disorder [F25.x], delusional disorders [F22.x, F24], unspecified nonorganic psychosis (F28/F29), psychotic disorders due to psychoactive substance use ([F10-F19].5 and [F10-F19].7), and affective psychoses (mania with psychotic symptoms [F30.2], bipolar affective disorder with psychotic symptoms [F31.2, F31.5], and depression with psychotic symptoms [F32.3/F33.3]). Differing from the SLaM model (12), F23.x codes (acute and transient psychotic disorders) were not included in the outcome diagnoses.

### 2.8 Expansion to NLP-specified diagnosis

In the mental health service CRIS dataset to 23rd April 2023, there were data on 430,746 patients. Only 39,771 had any F-code ICD-10 diagnosis recorded in structured fields, and fewer had a relevant baseline diagnosis meeting our inclusion criteria. We discovered that this was because the ICD-10 structured fields were generally only recorded when patients were admitted to an inpatient setting.

Therefore, in ordered to reduce this bias, diagnoses extracted by the NLP “NER + context classification” model were also used to identify diagnosis from case notes.

### 2.9 Model specifications

All model analyses were run in R (version 4.3.1). All individuals who received an index diagnosis of an ICD-10 non-organic, non-psychotic disorder, and had gender, age, and ethnicity recorded were eligible. A Cox proportional hazards model was used to predict the hazard ratio of developing a psychotic disorder over time. The predictors of the original model included: age (at the time of index diagnosis), gender, age by gender interaction, ethnicity, and cluster index diagnosis as described above and shown in **Supplementary Table 2** (weights used for each predictor were drawn from the Fusar-Poli et al’s study (2),). The NLP-refined model additionally considered the presence or absence of the 14 NLP-derived symptom and substance use predictors (tearfulness, poor appetite, weight loss, insomnia, cannabis use, cocaine use, guilt, irritability, delusions, hopelessness, poor insight, agitation, paranoia, disturbed sleep and insomnia) in the six months prior to index diagnosis, extracted by mention only (no context classification). The model was run applying the weights to each predictor that were derived by Irving et al., in their 2021 study (12); these are shown in **Supplementary Table 3**.

### 2.10 Assessing performance of the original and NLP-refined transdiagnostic risk calculator in Southeast England data

Validation and reporting were conducted in accordance with the best practice recommendations (21–23) and the Transparent Reporting of a multivariable prediction model for Individual Prognosis Or Diagnosis (TRIPOD-AI) guidelines (24).

We described the differences between Southeast England and SLaM datasets reported in the Irving et al. study (12). Baseline clinical and sociodemographic characteristics of the Southeast England and SLaM samples (including missing data) were described by means and standard deviations (SDs) for continuous variables, and absolute and relative frequencies for categorical variables.

We then visually compared the Kaplan–Meier failure functions of the Southeast England and SLaM datasets. The overall cumulative risk of psychosis onset in Southeast England was visualized with the Kaplan–Meier failure function (1—survival) and 95% confidence intervals (CIs). Curves that vary noticeably may indicate systematic differences within the study populations (22).

We calculated the predicted probabilities for each participant with complete predictor data in the Southeast England dataset (i.e. complete case analysis) from the regression coefficients obtained from the NLP-refined model developed in the SLaM dataset (**Supplementary Table 3**). The Prognostic Index (PI) for an individual can be understood as the log relative hazard (based on the sum of the regression coefficients of the variables in the model) from the Cox regression, compared with a hypothetical individual whose PI is zero (22).

We then sought to provide evidence that the model could distinguish between individuals with and without the outcome of interest (discrimination); produce risk estimates that have good agreement with observed risk (calibration); and show potential net benefit over other approaches (clinical utility) (6). Discrimination was assessed using the Harrell’s Concordance-index (C) (25), a widely used metric in survival analysis (with time-to-event outcomes), to assess the discriminatory power of a prognostic model. It quantifies the model’s ability to correctly rank individuals based on their predicted risk of experiencing a specific event over time. Mathematically, the C-index is computed as the proportion of concordant pairs among all comparable pairs. It ranges from 0.5 to 1.0. A value of 1.0 signifies perfect discrimination, where the model consistently and accurately ranks individuals according to their observed event times, 0.9-0.99 outstanding discrimination, 0.8-0.89 excellent, 0.7-0.79 acceptable, while a score of 0.5 indicates performance at chance. We estimated overall model performance using the Brier score (the average mean squared difference between predicted probabilities and actual outcomes, which also captures calibration and discrimination aspects; 0=most accurate; 1=least accurate) (26).

Calibration was assessed using the regression intercept (0=optimal) and slope (1=perfect agreement) of the PI (22). Decision curve analysis (27) was used to assess clinical utility by estimating net benefit across a range of feasible thresholds (i.e., the risk score at which an intervention would be deemed necessary). Classical decision theory proposes that at a chosen risk threshold, the choice with the greatest net-benefit should be preferred (28).

### 2.11 Sensitivity analyses

We repeated discrimination, calibration and clinical utility analyses only including structured ICD-10 diagnosis data.

To investigate the maximum possible best fit of the model, we ran a Cox regression with LASSO regularization, training this on 80% of the data and holding out 20% for testing the trained model.

## 3 Results

### 3.1 NLP algorithms

As shown in **Table 1**, the NER model achieved our target F1 score of >80% on all target concepts, although F1 score on individual symptoms was not able to be calculated.

**Table 1:** Precision, recall and F1 scores for all NER concepts.

Concept	Training set count	Validation set count	Test set count	Precision	Recall	F1
medication	2651	526	529	0.97	0.95	0.96
dosage	1257	238	236	0.97	0.98	0.97
diagnosis	1767	374	354	0.75	0.92	0.83
patient experience	7349	1546	1525	0.79	0.87	0.82
substance use	936	189	182	0.85	0.92	0.88
Average				0.86	0.93	0.89

The performance of the context classification model for diagnosis was assessed on a test set of 100 sentences for each of the implicit fields. There were four possible categories in Status (“has”, “had, “does not have”, “could have”) and two possible categories in Experiencer (“patient” and “other”); 600 test sentences in total. The performance achieved on this test averages an F1-score of 0.90 meeting our performance target, as shown in **Table 2**.

**Table 2:** Performance of contextual classification model for diagnosis.

Implicit field	Implicit field value	Precision	Recall	F1
Status	Has	0.93	0.80	0.86
	Had	0.88	0.96	0.91
	Does not Have	0.91	0.98	0.94
	Could Have	0.87	0.86	0.87
Experiencer	Patient	0.99	0.94	0.96
	Other	0.94	0.99	0.96

### 3.2 Southeast England sample characteristics

Data were extracted on 12^th^ April 2023.

**Figure 1:**
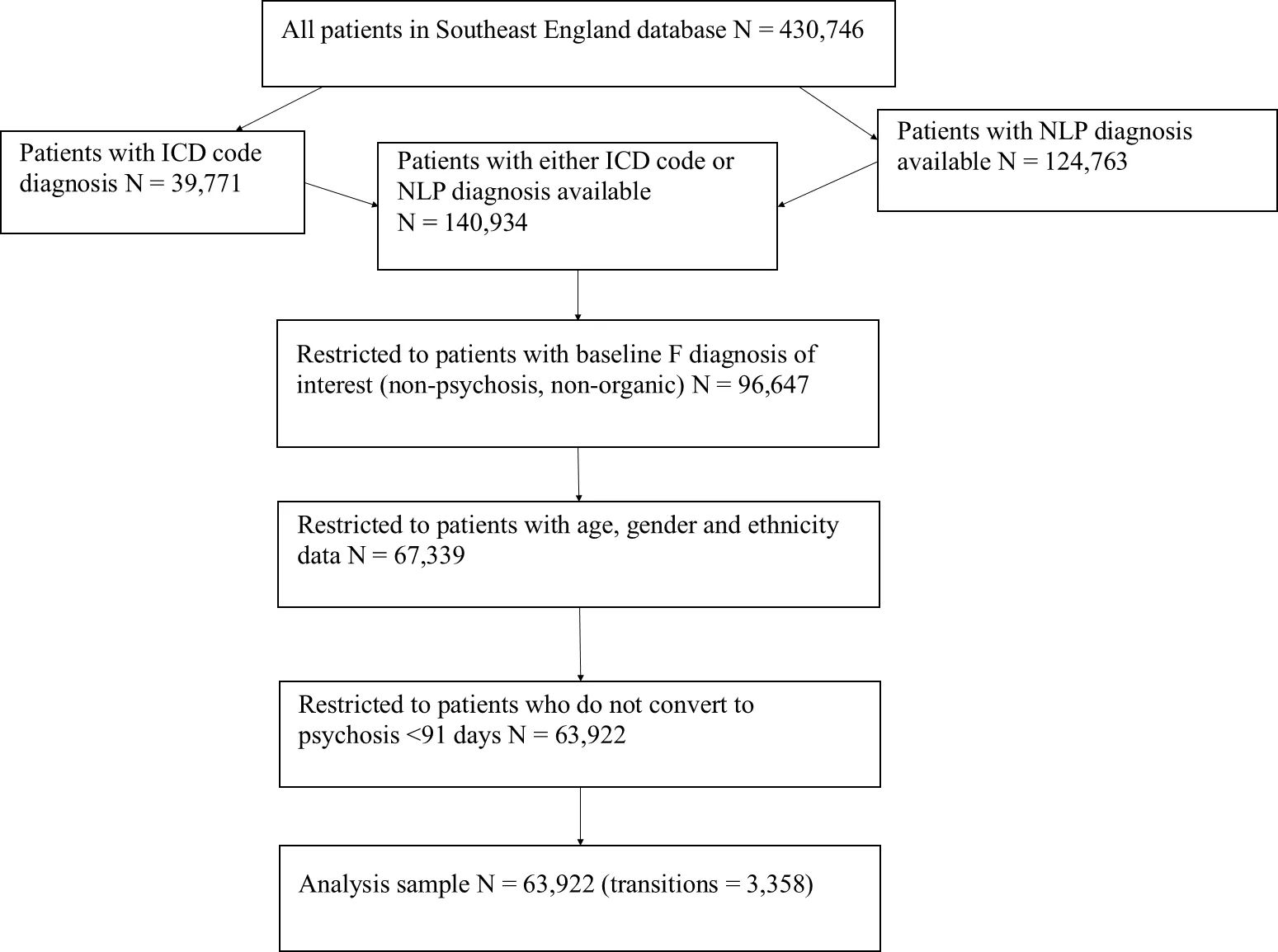
Flowchart detailing patient selection for analysis.

140,934 patients were identified with an ICD-code or NLP-derived diagnosis or both (124,763 patients (approximately 29% of the total sample) had a baseline F diagnosis derived by NLP). Of these, 96,647 had a diagnosis of interest at baseline (non-psychosis, non-organic). We then restricted the sample to those who additionally had age, gender and ethnicity data available (N=67,339). Once the patients who had a new psychosis diagnosis within 91 days of baseline were removed, our final sample was 63,922 patients, of whom 3358 transitioned to psychosis later than 91 days after baseline (See **Figure 1**).

**Figure 2:**
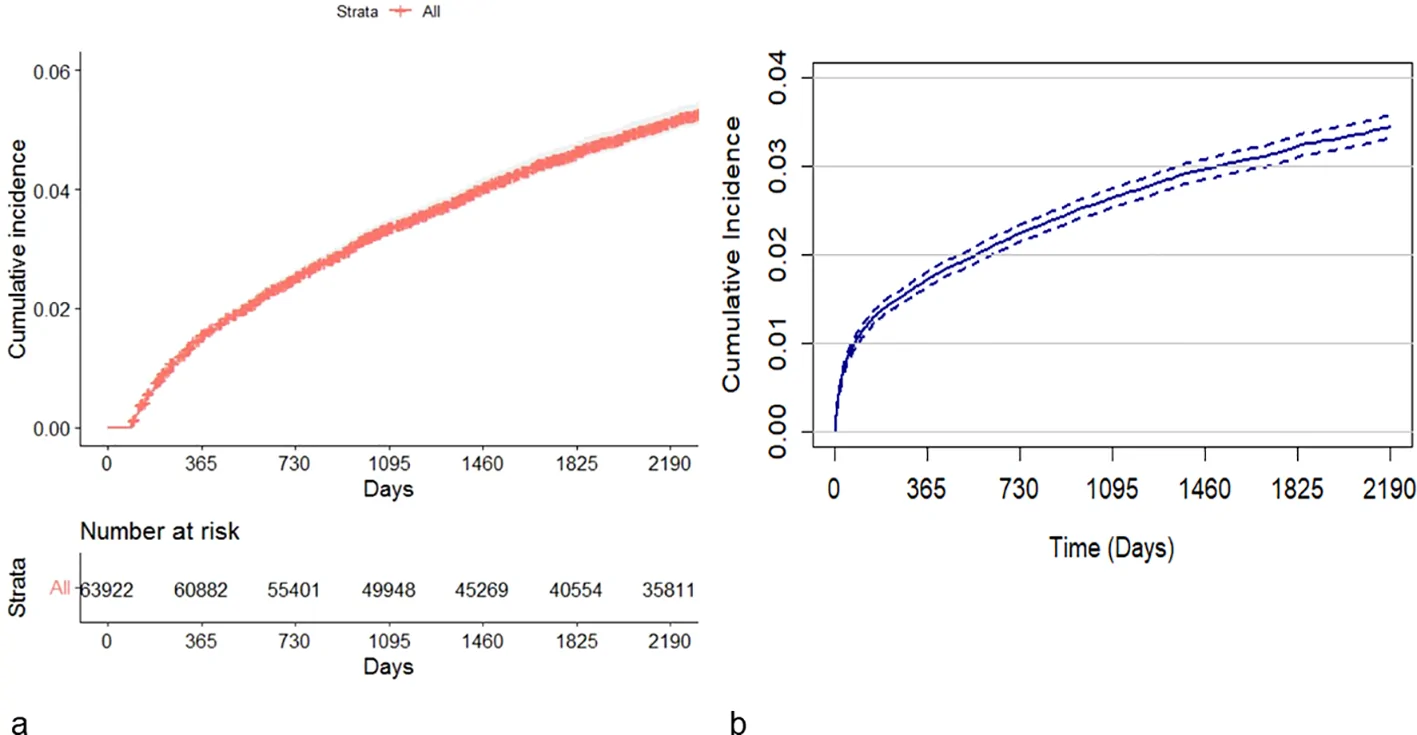
Cumulative incidence (Kaplan–Meier failure function) for risk of development of psychosis in **(a)** Southeast England (n=63,922) and in **(b)** SLaM (n=92,151). Time on the figure is presented in days. **(a)**: The cumulative incidence of psychosis was 1.48 (95%CI: 1.45-1.64, 60,882 individuals still at risk) at 1 year, 2.51 (95%CI: 2.38-2.63, 55,401 individuals still at risk) at 2 years, 3.35 (95%CI: 3.20-3.49, 49,948 individuals still at risk) at 3 years, 4.00 (95%CI: 3.84-4.16, 45,270 individuals still at risk) at 4 years, 4.59 (95%CI: 4.42-4.77, 40,555 individuals still at risk) at 5 years, and 5.12 (95%CI: 4.93-5.31, 35,811 individuals still at risk) at 6 years. The last transition to psychosis was at 2,183 days when 35,814 individuals were still at risk. **(b)**: 92,151 patients accessing SLaM during 1st Jan 2008 - 28th July 2018. There were 1556 events in the first 365 days, 418 events in the interval 365-730 days, 294 events in the interval 730-1095 days, 204 events in the interval 1095-1460 days, 150 events in the interval 1460-1825 days, 100 events in the interval 1825- 2190 days. The last failure was observed at 2190 days, when 41108 patients were still at risk.

For the full sample of patients with either ICD-code or NLP-derived baseline diagnosis, as well as data on gender, age and ethnicity (N = 63,922), the median age was 24 years [IQR 15-44], 49% were female, 95% had white race/ethnicity recorded, 1.2% had Asian ethnicity recorded, 0.9% had Black ethnicity recorded, 2.3% had mixed ethnicity recorded, and 0.8% had “Other” ethnicity recorded (**Table 3**). The median follow-up for the whole sample was 2476 days (range 91-8674; IQR 1258-3510). For the patients who received a psychosis diagnosis during follow up (and were therefore censored at the time point of this diagnosis) the median follow-up time to transition was 913 days (range 91-8674, IQR 334-1917) (**Figure 2**).

**Table 3:** Baseline sociodemographic and clinical characteristics.

Characteristic	Southeast England N=63,922	Irving full study population N=92150
Age (years)	Mean 30.8 (SD 20.4)	Mean 33.6 (SD 19.0)
Sex Male	32337 (51%)	45410 (49.3%)
Sex Female	31545 (49%)	46741 (50.7%)
Index Diagnosis		
Acute and transient psychotic disorders	479 (0.7%)	1568 (1.7%)
Substance Use Disorders	2521 (3.9%)	16754 (18.2%)*
Bipolar Mood Disorders	5561 (8.7%)	3149 (3.4%)
Non-Bipolar Mood Disorders	7537 (12.0%)	15965 (17.3%)
Anxiety Disorders	11165 (17%)	25323 (27.5%)
Personality Disorders	3932 (6.2%)	3524 (3.82%)
Developmental Disorders	17345 (27%)	4645 (5.04%)
Childhood/adolescence-onset Disorders	12781 (20%)	12332 (13.4%)
Physiological Syndromes	2544 (4.0%)	6806 (7.39%)
Intellectual disabilities	57 (<0.1%)	1640 (1.78%)
Ethnicity		
Asian	742 (1.2%)	4549 (4.94%)
Black	558 (0.9%)	15187 (16.5%)
Mixed	1456 (2.3%)	3805 (4.13%)
Other	484 (0.8%)	6899 (7.49%)
White	60682 (95%)	61711 (67.0%)
NLP signs and symptoms		
*Cocaine*	1900 (3%)	10229 (11.1%)
*Lack of Insight*	32 (<0.1%)	17089 (18.5%)
*Paranoia*	3614 (5.7%)	13212 (14.3%)
*Tearfulness*	10622 (17%)	20214 (21.9%)
*Loss of appetite*	938 (1.5%)	13653 (14.8%)
*Weight loss*	2069 (3.2%)	8623 (9.36%)
*Insomnia*	1237 (1.9%)	5115 (5.55%)
*Guilt*	2423 (3.8%)	9953 (10.8%)
*Irritation*	1316 (2.1%)	9049 (9.82%)
*Delusion*	2519 (3.9%)	5352 (5.81%)
*Hopelessness*	3405 (5.4%)	8883 (9.64%)
*Disturbed Sleep*	1482 (2.3%)	25786 (28.0%)
*Agitation*	3405 (5.3%)	12916 (14.0%)
*Cannabis*	3957 (6.2%)	13604 (14.8%)

*Discrepancy due to absence of substance use services in Southeast England Trust.

### 3.3 Transdiagnostic risk calculator performance in Southeast England data

Using structured ICD-10 diagnoses only, there were 11,724 patients with a baseline predictor diagnosis as specified in the original model, and 953 of these had a subsequent diagnosis of psychosis. Using the same weightings as were used in the original model (for age, gender, ethnicity and primary diagnosis), we found a concordance score (Harrell’s C) of 0.645 (SE=0.01). When adding in the available NLP predictors to this sample, the model showed a concordance score of 0.705 (SE=0.009).

Using NLP-derived plus structured ICD-10 diagnoses, there were 63,922 patients with a baseline predictor diagnosis as specified in the original model, and 3,558 of these were subsequently diagnosed with psychosis. Using the same weightings as were used in the original model (for age, gender, age by gender, ethnicity and primary diagnosis), we found a concordance score (Harrell’s C) of 0.671 (SE=0.005) and Brier score of 0.05 at six years. Calibration plots showed evidence of miscalibration with over-estimation of psychosis risk at lower predicted probabilities and under-estimation of psychosis risk at higher predicted probabilities (intercept = 1.41, slope = 1.61; **Supplementary Figure 1A**). When the NLP-derived predictors were added, the concordance score increased to 0.707 (SE=0.005) and Brier score remained at 0.05. Median PI was -1.82 with a range of -3.17 to 3.1 (**Supplementary Table 4**). Calibration plots showed good agreement between observed and estimated risk at most predicted probabilities with some evidence of under-estimation of psychosis risk at higher predicted probabilities (intercept = -0.12, slope = 1.04; **Supplementary Figure 1B**).

### 3.4 Sensitivity analyses

With a Cox regression model, using LASSO to optimize the predictor set and their weights in this new data, concordance reached 0.73 in the training dataset, and 0.66 in the held-out test data, showing no substantial improvement over the Irving et al. model (12) (weights shown in **Supplementary Table 5**).

## 4 Discussion

We have reported work to evaluate the local adaptation of a well-established English risk calculator for first episode psychosis in Southeast England mental health data. We have identified and overcome a number of barriers in replicating the calculator developed in SLaM, and trialled some adaptations, such as using NLP-enriched baseline and outcome diagnosis in the Southeast England dataset. Our replication identified a concordance statistic of around 0.71 which compares favorably with the other replications of this same calculator in different populations and datasets.

While several teams have produced calculators to identify patients at clinical high risk for psychosis (CHR-P) (29), the SLaM calculator is the only such tool known to the authors which is entirely populated from elements found in the patients’ EHR. Our replication work shows the calculator compares favorably to other tools which use more resource intensive data collection. Tools in North America, Australia, China and Korea have achieved a predictive accuracy in the development cohort of C-index 0.71-0.78 and external validation accuracy of AUC 0.79-0.80, but have used subscales of clinical assessments and functional evaluations rather than routinely collected clinic data (30–35). Performance of such tools in non-differentiated patients in primary care settings and community secondary mental health care settings (outside of specialized high risk services) has not yet been fully investigated (29), and few tools have been replicated in novel settings. The SLaM risk calculator has been successfully replicated in a number of new settings such as elsewhere in London (10), Oxford, UK (11), and the USA (13), retaining an acceptable performance (Harrell’s C range 0.73-0.79 in UK samples, dropping to 0.68 in US). Notably these tended to be more urban settings than the predominantly rural and coastal counties in this study, in which the population mainly lives in villages and small to medium size towns. Thus, the replication of the calculator maintaining a concordance of over 0.70 suggests that it is robust to such population demographic differences.

### 4.1 Strengths and limitations

Our study was not able to replicate the SLaM calculator identically in the Southeast England dataset, instead the calculator development required several modifications. It is difficult to say whether a loss of accuracy in the Southeast England risk calculator would be due to differences in the Southeast England population, or differences in the way Southeast England data was captured and processed. A further major difference between the two calculators was that the original SLaM NLP algorithms were largely rule-based, whereas the Akrivia algorithms were based on machine learning (ML) and open-source transformer architecture. The iterations to optimize these ML models took time, and the Akrivia team were able to update the Southeast England dataset numerous times with newer and more accurate NLP model outputs, the near final versions of which were used for this analysis. Despite the effort and time spent in developing novel NLP, we found strong discrepancies in the frequency of the different signs and symptoms used as predictors in the calculator between our data and that reported in the SLaM dataset (12), and while at least some of these differences are due to variances in services, recording style and patient populations, some may be attributed to the differing NLP approaches. Further investigation to identify the influence of NLP approach to concept capture and frequency is warranted.

A second replication barrier was that the original model developed in SLaM data relied on structured diagnostic data, both for the baseline diagnosis and for the predicted outcome (psychosis diagnosis). We found in the Southeast England dataset, only 8.8% of patients had any ICD diagnostic code and only 3.7% of our sample had a relevant structured diagnosis (ICD code) at baseline. It was likely that those patients who received an ICD code at baseline differed systematically from those who did not (i.e. were inpatients admitted to psychiatric hospitals), and we wanted our model to be applicable to community-dwelling patients. We chose to include NLP-derived diagnoses in our model which deviated from the SLaM approach, meaning that the current presented calculator is an adapted version rather than a true replication. We noted that the models achieved similar accuracy in the NLP-diagnosis dataset to including structured diagnosis-only, but still only a proportion of patients had an NLP-derived diagnosis in their record, the rest had to be excluded. It is not clear what implications the inclusion of NLP-derived diagnosis will have on the use of the model in clinical settings, as we acknowledge that NLP diagnosis algorithms are not 100% accurate in identifying true diagnostic data in text. A further issue for NLP development for extracting diagnosis was that we expected the most common occurrence of diagnostic information outside of structured fields to be in semi-structured formats in letters and reports. These letters and reports were not available in the Akrivia dataset at the time of our study. Future research will examine if these make a substantial difference to extraction of diagnostic information or to the sample of patients who could be included in the analysis.

We identified demographic differences in the Southeast England dataset compared to the original SLaM sample. Although our sample had a broadly similar median age and gender split, the proportion of White ethnicity in the Southeast England sample was far higher than in the London sample (95% vs 61%) and a large proportion of ethnicity in Southeast England data was recorded as “Other” or was missing; these patients had to be excluded from the analysis sample. Many patients’ data was excluded from the analysis because of missing demographics; we experienced a 30% loss in numbers when we applied the criterion that age, ethnicity and gender must be present. Our model will not generalize to those patients for whom basic demographic data is missing, and these patients may, again, differ systematically to those for whom higher quality data is captured. There were also differences in service configuration, with no early detection services for psychosis or substance use services present in Southeast England, while the Southeast England data contained neurodevelopmental pathway data (for e.g. autism diagnosis) that SLaM does not have. SLaM has a specific treatment center for anxiety and trauma, which is not present in Southeast England. It also seems likely that there were other service variations which were reflected in differences in diagnostic prevalence between the two samples. Despite these differences in case-mix and service configuration, the calculator performed broadly similarly to its other replications in novel settings suggesting it may be robust to these differences.

### 4.2 Future work

Due to the differences in the Southeast England dataset compared to those of the SLaM development dataset, the final Southeast England risk calculator must run on more NLP-derived clinical data than the SLaM model did. We note that the field of NLP is rapidly changing and improving with the advent of highly accurate large language models, therefore the Akrivia team continually spends time refining, testing and augmenting NLP algorithms in order to ensure high accuracy, and replication of such risk calculators may therefore show higher accuracy in the future. In addition, the team hope to apply the NLP algorithms to letters and reports, rather than just the case notes, which is expected to increase the volume and accuracy of diagnostic NLP data.

Public and Patient Involvement (PPI) was a key part of the study, with a PPI lead as a co-applicant and co-author. The PPI role was very important in terms of ensuring a technical study was understandable, ethical and translatable to possible end users. We engaged closely with a lived-experience advisory panel formed of people who have direct experience of mental health and psychosis. Older panel members were concerned about the accuracy of the NLP algorithms and wanted to ensure that the application of the calculator would be followed by a clinical review to ensure that people were not identified as ‘at risk’ inappropriately. Younger people [from a Youth PPI café (36)] liked the fact that the calculator would be applied in the same way for everyone, reducing the risks of clinician bias, and were keen on the prospect that the calculator may be able to provide unique individual risk profiles. Both groups liked the fact that the calculator might reduce the risk of people ‘falling through gaps’ between services. People also wanted to ensure that the ‘risk state’ was conveyed appropriately, bearing in mind that only a proportion of flagged patients will go on to develop psychosis, with hopeful and non-stigmatizing language. The role and impact of PPI will be further explored in an additional paper.

### 4.3 Clinical implications

At the present time our model has achieved a best accuracy (measured by Harrell’s C) of 0.71 using SLaM model weights. It is not clear if the prognostic potential of this model will be of clinical value when applied prospectively to current patients using the Southeast England healthcare system. We are currently conducting evaluation activities during a pilot phase of the risk calculator implementation, including careful clinical review of identified high-risk patients. In addition to the calculator, we have co-designed psychoeducation, intervention and care pathway information, and developed a training video for use with staff in secondary, primary and third sector organizations, including consultations and co-production with patients, clinicians, service leads and commissioners. A feasibility trial of the prospective identification of patients at high risk using the risk calculator is underway, assessing the implementation and uptake of the co-designed targeted mental health support in the Southeast England population.

### 4.4 Conclusion

Refining and replicating a risk calculator for psychosis, in order to implement it in the health system of a new geographical area, with different population demographics, and non-comparable clinical data, could be problematic. We show it is achievable where there is willingness to adapt to novel data in the new setting, however the risk calculator may differ substantially from the original and require additional validation. We aim to pilot and carefully evaluate the implementation of the risk calculator within the Southeast England mental healthcare system, in order to identify people at risk of psychosis, and to offer them tailored intervention packages to reduce their risk of serious health outcomes.

## Data Availability

The data analyzed in this study is subject to the following licenses/restrictions: All study data is carefully protected in a secure data environment and cannot be made publicly available. Analytical code can be accessed by contacting the study authors via primarycareda@bsms.ac.uk. Requests to access these datasets should be directed to Contact@akriviahealth.com.
