# Correction: Local adaptation and validation of a transdiagnostic risk calculator for first episode psychosis using mental health patient records

**DOI:** 10.3389/fpsyt.2026.1993411

**Published:** 2026-09-18

**Authors:** Elizabeth Ford, James Stone, Dominic Oliver, Benjamin Fell, Gloria Roque, Sam Robertson, Paolo Fusar-Poli, Kathryn Greenwood

**Affiliations:** 1 Department of Primary Care and Public Health, Brighton and Sussex Medical School, University of Brighton and University of Sussex, Brighton, United Kingdom; 2 Department of Clinical Neuroscience, Brighton and Sussex Medical School, University of Brighton and University of Sussex, Brighton, United Kingdom; 3 Sussex Partnership National Health Service (NHS) Foundation Trust, Worthing, United Kingdom; 4 Department of Psychiatry, University of Oxford, Oxford, United Kingdom; 5 National Institute of Health Research (NIHR) Oxford Health Biomedical Research Centre, Oxford, United Kingdom; 6 OPEN Early Detection Service, Oxford Health NHS Foundation Trust, Oxford, United Kingdom; 7 Akrivia Health, Oxford, United Kingdom; 8 Department of Brain and Behavioral Sciences, University of Pavia, Pavia, Italy; 9 Early Psychosis: Interventions and Clinical-Detection (EPIC) Lab, Department of Psychosis Studies, Institute of Psychiatry, Psychology and Neuroscience, King’s College London, London, United Kingdom; 10 OASIS Service, South London and Maudsley NHS Foundation Trust, London, United Kingdom; 11 Department of Psychiatry and Psychotherapy, Ludwig-Maximilian-University, Munich, Germany; 12 School of Psychology, University of Sussex, Brighton, United Kingdom

**Keywords:** psychosis, risk assessment, natural language processing, electronic health records, mental health care, at risk mental state

Affiliation 1, “Department of Primary Care and Public Health, Brighton and Sussex Medical School, University of Brighton and University of Sussex, Brighton, United Kingdom”, was erroneously captured as “Department of Primary Care and Public Health, Brighton and Sussex Medical School, Brighton, United Kingdom” for author “Elizabeth Ford”.

Affiliation 2, “Department of Clinical Neuroscience, Brighton and Sussex Medical School, University of Brighton and University of Sussex, Brighton, United Kingdom”, was erroneously captured as “Department of Clinical Neuroscience, Brighton and Sussex Medical School, Brighton, United Kingdom” for author “James Stone”.

Author “James Stone” was erroneously affiliated with “School of Psychology, University of Sussex, Brighton, United Kingdom”. This affiliation has been removed for author “James Stone”.

The original version of this article has been updated.

