# Supplementary material for: Local adaptation and validation of a transdiagnostic risk calculator for first episode psychosis using mental health patient records
Source: Front Psychiatry. 2025 Jul 22;16:1584719. doi: 10.3389/fpsyt.2025.1584719 (PMC12323713; doi:10.3389/fpsyt.2025.1584719)
Supplement: Supplementary file 1 [file SupplementaryFile1.docx]

**Supplementary Table 1:** Concepts used in the regression model

| **Concepts – Original Structured Model** | **New Akrivia NLP Terms and Concepts** |
| --- | --- |
| Baseline diagnosis | From structures data and NLP derived diagnoses mapped to ICD codes outlined below |
| Sex | (from structured data) |
| Age | (from structured data) |
| Ethnicity | (from structured data) |
| **Concepts – Original Model NLP variables** |  |
| emotion – tearfulness | Crying |
| appetite – loss | Loss of appetite, decrease of appetite, finding of appetite, Altered appetite, Appetite normal |
| appearance – weight loss | Overweight, Underweight, Weight gain, Weight loss, Weight steady |
| thoughts – guilt | Feeling guilt |
| emotion – irritability | Feeling irritable |
| thoughts – delusion | Delusion of persecution, Delusions, Paranoid delusions |
| thoughts – hopelessness | Feeling hopeless |
| Poor insight | Impaired insight, Lack of insight |
| agitation | Feeling agitated, Hyperactive behaviour, Feeling excited |
| thoughts – paranoia | Paranoid symptoms |
| Disturbed sleep | Disturbed Sleep |
| Insomnia | Insomnia |
| Cocaine use | Substance use type-  cocaine |
| Cannabis use | Substance use type-  cannabis |
| **Concept – Outcome to be predicted** |  |
| Psychosis diagnosis/psychotic episode. | From Structured data and NLP derived diagnoses mapped to ICD codes outlined below |

**Supplementary Table 2**: ICD Codes used to define primary index diagnosis (taken from Irving et al., 2021 [12])

| **Primary index diagnosis** | **ICD-10 code** | **ICD-10 diagnosis name** |
| --- | --- | --- |
| Acute and transient psychotic disorders | F23.x | Acute and transient psychotic disorders |
| Substance use disorders | F10 (excluding *.5, *.4, *.7) | Nonpsychotic mental and behavioural disorders due to use of alcohol |
|  | F11 (excluding *.5, *.4, *.7) | Nonpsychotic mental and behavioural disorders due to use of opioids |
|  | F12 (excluding *.5, *.4, *.7) | Nonpsychotic mental and behavioural disorders due to use of cannabinoids |
|  | F13 (excluding *.5, *.4, *.7) | Nonpsychotic mental and behavioural disorders due to use of sedatives or hypnotics |
|  | F14 (excluding *.5, *.4, *.7) | Nonpsychotic mental and behavioural disorders due to use of cocaine |
|  | F15 (excluding *.5, *.4, *.7) | Nonpsychotic mental and behavioural disorders due to use of other stimulants, including caffeine |
|  | F16 (excluding *.5, *.4, *.7) | Nonpsychotic mental and behavioural disorders due to use of hallucinogens |
|  | F17 (excluding *.5, *.4, *.7) | Nonpsychotic mental and behavioural disorders due to use of tobacco |
|  | F18 (excluding *.5, *.4, *.7) | Nonpsychotic mental and behavioural disorders due to use of volatile solvents |
|  | F19 (excluding *.5, *.4, *.7) | Nonpsychotic mental and behavioural disorders due to multiple drug use and use of other psychoactive substances |
| Bipolar mood disorders | F31.x (excluding F31.2 and F31.5) | Nonpsychotic bipolar disorder |
|  | F34.0 | Cyclothymia |
|  | F30.x (excluding *.2) | Nonpsychotic mania or hypomania |
| Non bipolar mood disorders | [F32-F33].x (excluding F32.3 and F33.3) | Nonpsychotic depressive disorder |
|  | F34.1 | Dysthymia |
|  | F34.8, F34.9, F38.x, F39 | Unspecified mood disorders |
| Anxiety disorders | F40.x | Phobic anxiety disorders |
|  | F41.0 | Panic disorder |
|  | F41.1 | Generalized anxiety disorder |
|  | F41.2-F41.9 | Other anxiety disorders |
|  | F42.x | Obsessive compulsive disorders |
|  | F43.x | Reaction to severe stress, and adjustment disorders |
|  | F44.x | Dissociative [conversion] disorders |
|  | F45.x | Somatoform disorders |
|  | F48.x | Other neurotic disorders |
| Personality disorders | F60.0 | Paranoid personality disorder |
|  | F60.1 | Schizoid personality disorder |
|  | F60.2 | Dissocial personality disorder |
|  | F60.3 | Emotionally unstable personality disorder |
|  | F60.4 | Histrionic personality disorder |
|  | F60.5 | Anankastic personality disorder |
|  | F60.6 | Anxious [avoidant] personality disorder |
|  | F60.7 | Dependent personality disorder |
|  | F60.8-F60.9, F61, F62.x, F68.x, F69 | Other personality disorders |
|  | F21 | Schizotypal Disorder |
|  | F63.x | Habit and impulse disorders |
|  | F64.x, F65.x, F66.x | Sexual disorders |
| Developmental disorders | F80.x | Specific developmental disorders of speech and language |
|  | F81.x, F82, F83 | Other specific developmental disorders |
|  | F84.x | Pervasive developmental disorders |
|  | F88, F89 | Other and unspecified disorders of psychological development |
| Childhood/adolescence onset disorders | F90.x | Hyperkinetic disorders |
|  | F91.x | Conduct disorders |
|  | F92.x, F93.x, F94.x, F98.x | Other emotional and behavioural disorders with childhood or adolescence onset |
|  | F95.x | Tic disorders |
| Physiological syndromes | F50.x | Eating disorders |
|  | F51.x | Nonorganic sleep disorders |
|  | F52.x | Sexual dysfunction, not caused by organic disorder or disease |
|  | F53.x (excluding F53.1) | Non psychotic Mental and behavioural disorders associated with the puerperium, not elsewhere classified |
|  | F54.x, F55, F59 | Other physiological syndromes |
| Intellectual disabilities | F70.x | Mild Intellectual disabilities |
|  | F71.x | Moderate Intellectual disabilities |
|  | F72.x | Severe Intellectual disabilities |
|  | F73.x | Profound Intellectual disabilities |
|  | F78.x | Other Intellectual disabilities |
|  | F79.x | Unspecified Intellectual disabilities |
| *F00-F09 organic mental disorders and all psychotic disorders other than F23.x were excluded* | | |

**Supplementary Table 3** Regression weights applied to each predictor (taken from Irving et al., 2021 [12])

| **Concept** | **Beta Coefficient** |
| --- | --- |
| Sex (Male – relative to Female) | +0.251607368 |
| Age | +0.006559315 |
| Age*Sex(Male – relative to Female) | -0.007533502 |
| Ethnicity(Black – relative to White) | +0.768361197 |
| Ethnicity(Asian – relative to White) | +0.453691672 |
| Ethnicity(Mixed – relative to White) | +0.183875520 |
| Ethnicity(Other – relative to White) | +0.166521852 |
| Diagnosis(Acute and Transient Psychotic Disorders – relative to At Risk Mental State) | +0.231372239 |
| Diagnosis(Substance Use Disorders – relative to At Risk Mental State) | -1.907241596 |
| Diagnosis(Bipolar Mood Disorders – relative to At Risk Mental State) | -0.955396094 |
| Diagnosis(Non-Bipolar Mood Disorders – relative to At Risk Mental State) | -1.510699038 |
| Diagnosis(Anxiety Disorders – relative to At Risk Mental State) | -1.858113696 |
| Diagnosis(Personality Disorders – relative to At Risk Mental State) | -1.790252996 |
| Diagnosis(Developmental Disorders – relative to At Risk Mental State) | -2.705788876 |
| Diagnosis(Childhood-onset Disorders – relative to At Risk Mental State) | -2.836895336 |
| Diagnosis(Physiological Syndromes – relative to At Risk Mental State) | -2.240243726 |
| Diagnosis(Intellectual disabilities – relative to At Risk Mental State) | -2.657111217 |
| Cocaine Use mentioned in notes up to 6 months prior to index diagnosis | - 0.142318877 |
| Poor Insight mentioned in notes up to 6 months prior to index diagnosis | + 0.015966856 |
| Paranoia mentioned in notes up to 6 months prior to index diagnosis | + 0.961959253 |
| Tearfulness mentioned in notes up to 6 months prior to index diagnosis | -0.075196253 |
| Appetite loss mentioned in notes up to 6 months prior to index diagnosis | + 0.053663525 |
| Weight loss mentioned in notes up to 6 months prior to index diagnosis | + 0.129322246 |
| Insomnia mentioned in notes up to 6 months prior to index diagnosis | + 0.047588084 |
| Guilt mentioned in notes up to 6 months prior to index diagnosis | -0.070702784 |
| Irritability mentioned in notes up to 6 months prior to index diagnosis | + 0.051768711 |
| Delusions mentioned in notes up to 6 months prior to index diagnosis | + 0.739865889 |
| Feeling hopeless mentioned in notes up to 6 months prior to index diagnosis | -0.350144379 |
| Disturbed sleep mentioned in notes up to 6 months prior to index diagnosis | + 0.114071043 |
| Agitation mentioned in notes up to 6 months prior to index diagnosis | + 0.493122906 |

**Supplementary Table** **4:** PI values and cut offs

| **Prognostic Index: Estimated Values** |  |
| --- | --- |
| PI - range | -3.17 to 3.1 |
| Mean | -1.834 |
| Median | -1.85 |
| SD | 0.814 |
|  |  |
| **Cut offs for whole dataset:** |  |
| Top 10% | identify 649 with 227 transitions |
| Top 20% | 1264, 395 |
| Top 30% | 1915, 516 |
| Top 40% | 2558, 628 |


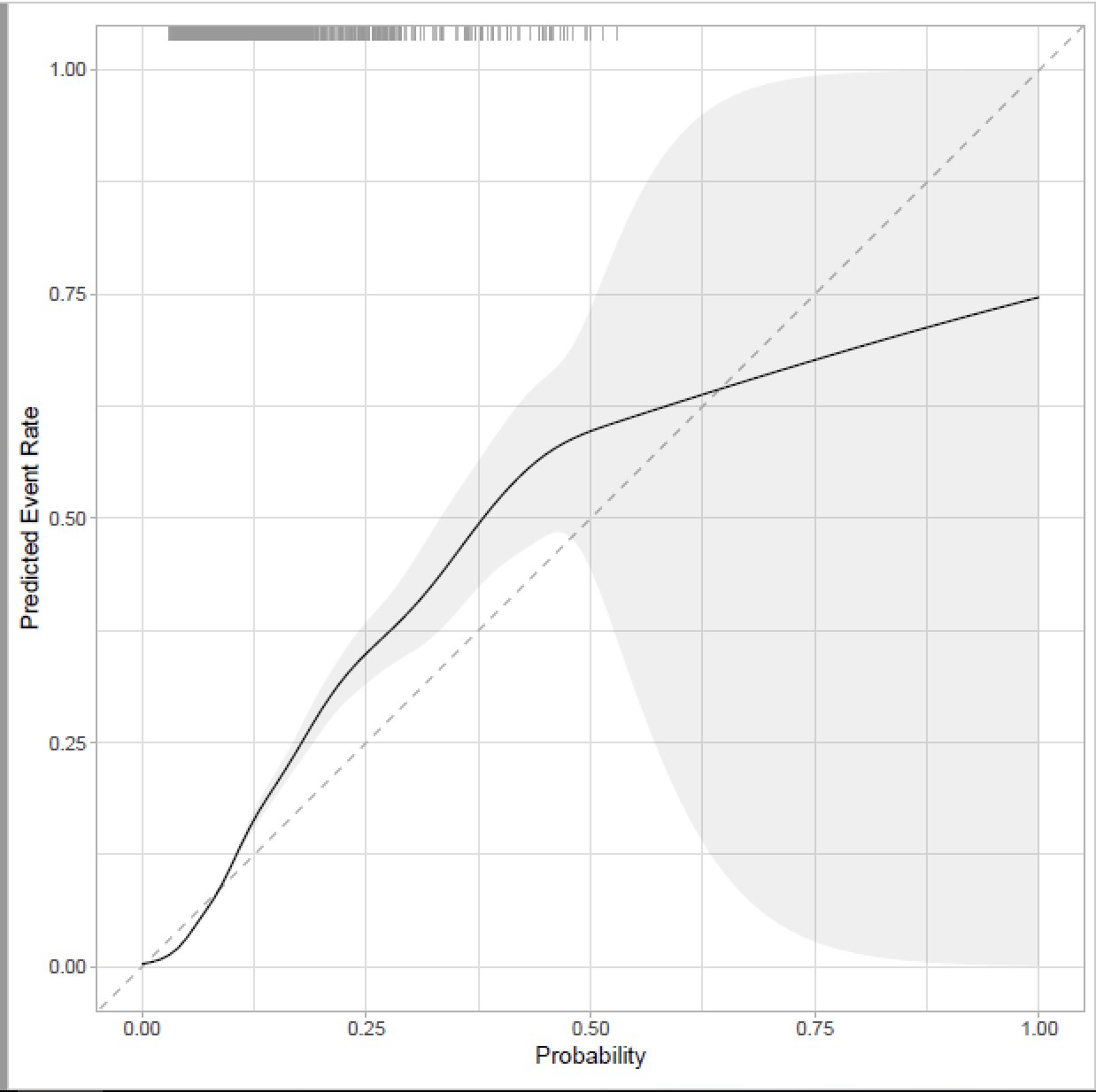

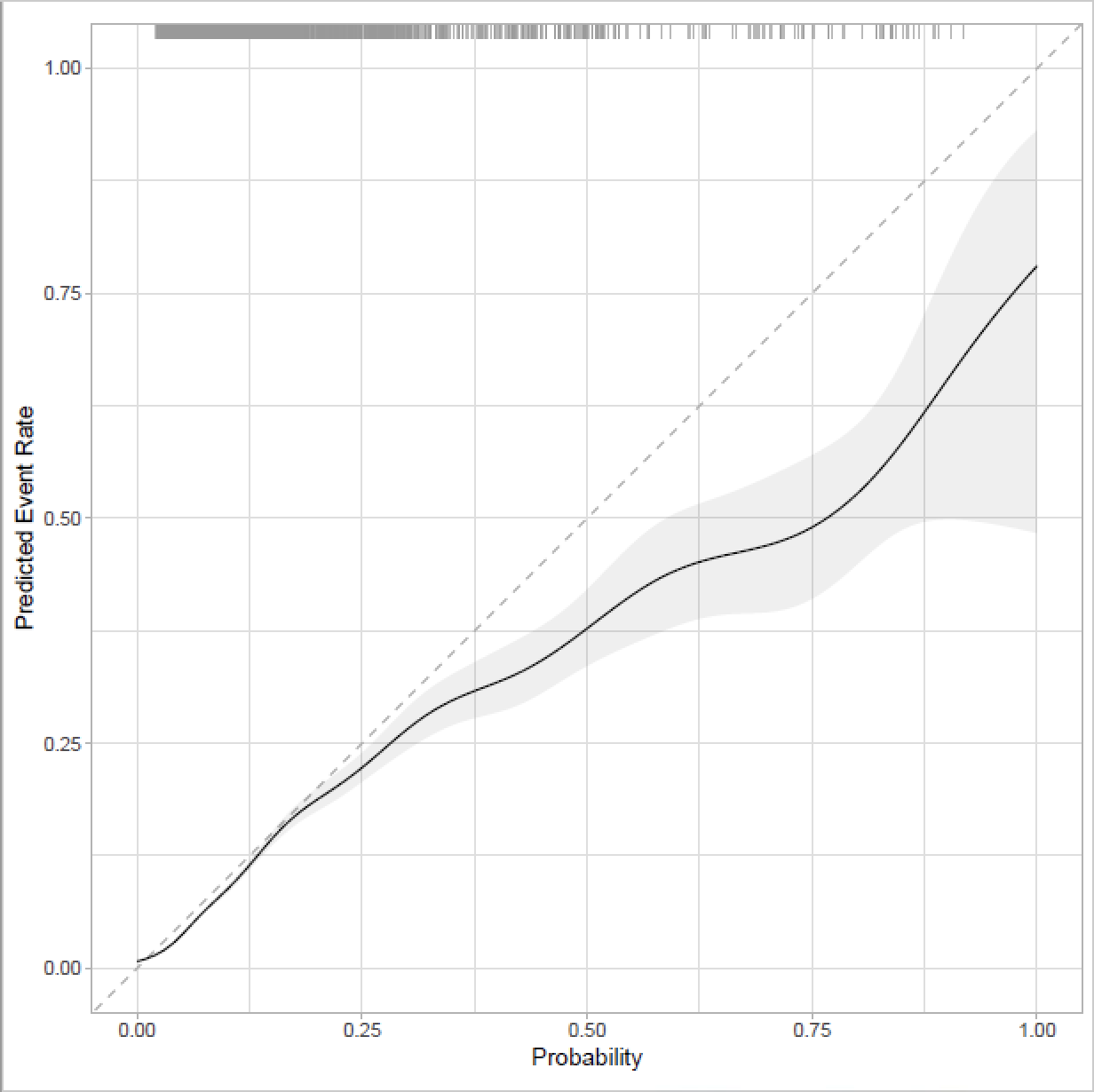
**Supplementary Figure 1** Calibration plots for original (A) and NLP-refined (B) transdiagnostic risk calculator for psychosis

**A**

**B**

**Supplementary Table 5** Comparison of Regression weights derived Irving et al. [12] and by LASSO from the Southeast England data

| **Concept** | **Irving et al.**  **Beta Coefficient** | **Southeast England**  **Lamda 1SE** |
| --- | --- | --- |
| Sex (Male – relative to Female) | +0.251607368 | -0.0629 |
| Age | +0.006559315 | +0.0109 |
| Age*Sex(Male – relative to Female) | -0.007533502 | Exc* |
| Ethnicity(Black – relative to White) | +0.768361197 | Exc |
| Ethnicity(Asian – relative to White) | +0.453691672 | Exc |
| Ethnicity(Mixed – relative to White) | +0.183875520 | Exc |
| Ethnicity(Other – relative to White) | +0.166521852 | Exc |
| Diagnosis(Acute and Transient Psychotic Disorders – relative to At Risk Mental State) | +0.231372239 | 1.4937 |
| Diagnosis(Substance Use Disorders – relative to At Risk Mental State) | -1.907241596 | Exc |
| Diagnosis(Bipolar Mood Disorders – relative to At Risk Mental State) | -0.955396094 | +0.6035 |
| Diagnosis(Non-Bipolar Mood Disorders – relative to At Risk Mental State) | -1.510699038 | Reference factor |
| Diagnosis(Anxiety Disorders – relative to At Risk Mental State) | -1.858113696 | Exc |
| Diagnosis(Personality Disorders – relative to At Risk Mental State) | -1.790252996 | +0.0709 |
| Diagnosis(Developmental Disorders – relative to At Risk Mental State) | -2.705788876 | Exc |
| Diagnosis(Childhood-onset Disorders – relative to At Risk Mental State) | -2.836895336 | -0.3052 |
| Diagnosis(Physiological Syndromes – relative to At Risk Mental State) | -2.240243726 | Exc |
| Diagnosis(Intellectual disabilities – relative to At Risk Mental State) | -2.657111217 | Exc |
| Cocaine Use mentioned in notes up to 6 months prior to index diagnosis | - 0.142318877 | Exc |
| Poor Insight mentioned in notes up to 6 months prior to index diagnosis | + 0.015966856 | Exc |
| Paranoia mentioned in notes up to 6 months prior to index diagnosis | + 0.961959253 | +0.8696 |
| Tearfulness mentioned in notes up to 6 months prior to index diagnosis | -0.075196253 | +0.0311 |
| Appetite loss mentioned in notes up to 6 months prior to index diagnosis | + 0.053663525 | Exc |
| Weight loss mentioned in notes up to 6 months prior to index diagnosis | + 0.129322246 | Exc |
| Insomnia mentioned in notes up to 6 months prior to index diagnosis | + 0.047588084 | Exc |
| Guilt mentioned in notes up to 6 months prior to index diagnosis | -0.070702784 | Exc |
| Irritability mentioned in notes up to 6 months prior to index diagnosis | + 0.051768711 | Exc |
| Delusions mentioned in notes up to 6 months prior to index diagnosis | + 0.739865889 | +0.4393 |
| Feeling hopeless mentioned in notes up to 6 months prior to index diagnosis | -0.350144379 | Exc |
| Disturbed sleep mentioned in notes up to 6 months prior to index diagnosis | + 0.114071043 | Exc |
| Agitation mentioned in notes up to 6 months prior to index diagnosis | + 0.493122906 | +0.1433 |
| Cannabis use mentioned in notes up to 6 months prior to index diagnosis | + 0.124130379 | +0.1003 |

*Exc: Predictors excluded by LASSO penalization.
